# Development of a scale to assess obsessive-compulsive tendencies among Japanese university students

**DOI:** 10.1016/j.heliyon.2022.e09646

**Published:** 2022-06-10

**Authors:** Kenta Sashikata, Eiji Ozawa

**Affiliations:** a Department of Graduate School of Human-Environment Studies, Kyushu University, 744, Motooka, Nishi-ku, Fukuoka, 819-0395, Japan; b Faculty of Human-Environment Studies, Kyushu University, 744, Motooka, Nishi-ku, Fukuoka, 819-0395, Japan

**Keywords:** Obsessive-compulsive tendencies, Obsessive-compulsive disorder, University students, Validity and reliability

## Abstract

Obsessive-compulsive (OC) tendencies refer to obsessions and compulsions in a nonclinical group, which are risk factors for obsessive-compulsive disorder (OCD). OC tendencies and OC symptoms are mainly assessed using five factors: ordering, obsessions, cleaning, hoarding, and checking. However, since hoarding is now classified as an independent diagnosis in the DSM-V, this factor was not included and was instead replaced by indecisiveness. Furthermore, many established scales used for measuring OC tendencies were originally created for OCD patients; thus, they cannot adequately capture OC tendencies. Therefore, this study aimed to develop a scale to assess OC tendencies among Japanese university students with a revised five-factor structure: ordering, obsessions, cleaning, indecisiveness, and checking. We examined the factor structure, reliability, criterion-related validity, and convergent validity of the OC tendencies scale by administering two surveys. In Survey 1 (*N* = 216), an exploratory factor analysis (EFA) was conducted to examine the criterion-related and convergent validity and reliability of the OC tendencies scale. In Survey 2 (*N* = 202), a confirmatory factor analysis (CFA) was conducted. EFA and CFA utilized a five-factor structure comprising checking, ordering, indecisiveness, cleaning, and obsessions. Correlations with other scales indicated that the OC tendencies scale had efficient convergent validity, criterion-related validity, internal consistency, and test-retest reliability. This study validated the five-factor structure of OC tendency in Japanese university students. However, indecisiveness was also strongly correlated with trait-anxiety. As this scale is easy to administer among large groups, it has the potential to contribute to mental health support for university students by measuring OC tendencies experienced on a daily basis, which have not been adequately measured in the past.

## 1 Introduction

Obsessive-compulsive disorder (OCD) is a severe mental health disorder characterized by obsessions and/or compulsions (American Psychiatric Association, 2013). The World Health Organization has identified OCD as one of the 10 leading causes of disability in the industrialized world (Murray and Lopez, 1996). In Japan, between 1996 and 2017, the number of patients with OCD increased from 8000 to 41000 (Japan Ministry of HealthLabour and Welfare, 2015, Japan Ministry of HealthLabour and Welfare, 2019). The psychological characteristics of OCD have been observed in non-clinical populations as well and have been termed as obsessive-compulsive (OC) tendencies in previous studies (Nicholson et al., 2014; Soref et al., 2008).

Pioneering research by Rachman and de Silva (1978) reported that unwanted thoughts similar in content and form to clinical obsessions are prevalent in the normal population. Moreover, “a vast majority of non-clinical individuals report having thought intrusions of sex, violence, dirt, and contamination” (Purdon and Clark, 1993, p. 718). Furthermore, a recent taxometric study examined the latent structure of obsessive-compulsive symptoms and related cognitions in an unselected sample of 1,005 student participants; that is, those who were not screened for the presence (or absence) of psychological disorders (Olatunji et al., 2008). Thus, prior studies support the notion that OC symptoms are dimensional in a continuous spectrum. Previous studies show that the factor structure of OC tendencies is similar to that of OCD (Sanavio and Vidotto, 1985; Sugiura and Tanno, 2000). Therefore, in this study, OC tendencies are defined as the psychological characteristics of obsessive-compulsive thoughts and behaviors that are common in the general population.

Although OC tendencies occur less frequently, cause less discomfort, and are shorter in duration than OCD symptoms, Li (2007) pointed out that people with high OC tendencies have trouble adapting to the environment and often experience ego dystonic symptoms regarding their obsessions and compulsions. Furthermore, OC tendencies are correlated positively with both suicidal ideation and an affinity for social withdrawal (Iliceto et al., 2017; Yasuda, 2019). Fukuda (2000) reported 12 cases of social withdrawal among Japanese university students, all with high OC tendencies and levels of perfectionism. Therefore, additional research on OC tendencies is needed to address the various psychological problems related to it.

Despite the ongoing controversy regarding the age of onset of OCD, de Lijster et al. (2017) showed that OCD emerges sometime between late adolescence and adulthood, based on a meta-analysis. University students face many difficulties, such as academic challenges and selecting a career, which is why they might be at a higher risk of developing OC tendencies and might require clinical interventions. Thus, additional research is needed to focus on OC tendencies among university students.

Many studies have examined the factor structure of OC tendencies and OCD (Bear, 1994; Leckman et al., 1997). Factor analytic studies related to OC symptoms were reviewed to reveal a similar four-factor structure: ordering, hoarding, cleaning, and obsessions (Mataix-Cols et al., 2005; Bloch et al., 2008). However, checking compulsions loaded the highest on the variables of obsessions in adults, whereas ordering was highest in children (Bloch et al., 2008). This difference may be due to the inherent ambiguity of the symptoms related to the checking category of the Yale-Brown Obsessive Compulsive Scale Symptom Checklist (Leckman et al., 2009). In light of the fact that many other scales measuring OC tendencies include the factor of checking (Sanavio, 1988; Hodgson and Rachman, 1977; Foa et al., 1998), it should be viewed as a single factor. Although the five-factor structure (ordering, obsessions, cleaning, hoarding, and checking) is considered reasonable, there are still two problems regarding the factor structure of OC tendencies and OCD.

First, the symptom of hoarding should not be used to measure OC tendencies because it is considered a symptom dimension of OCD. Hoarding is defined as “the acquisition of, and failure to discard, possessions which appear to be useless or of limited value” (Frost and Gross, 1993, p. 367). Moreover, in the past few years, an impressive number of studies have demonstrated that hoarding differs from other OCD. Bloch et al. (2014) showed that the presence of hoarding symptoms leads to poor treatment outcomes in OCD. Moreover, Hough et al. (2016) used functional magnetic resonance imaging to illustrate the differences in regional brain activation in individuals with hoarding symptoms and OCD, as well as in healthy groups. Wheaton, Timpano, LaSalle-Ricci, and Murphy (2008) revealed that OCD patients with hoarding had more severe OCD symptoms, and greater impairment and dysphoria, than OCD patients without hoarding. As a result, many researchers now consider hoarding to be a syndrome distinct from OCD (e.g., Abramowitz et al., 2008; Frost and Steketee, 2008; Rachman et al., 2009). Moreover, hoarding has been classified as a separate syndrome in the DSM-5, known as the hoarding disorder (American Psychiatric Association, 2013).

Second, indecisiveness should be considered to comprehensively measure OC tendencies experienced by university students. Indecisiveness is defined as a “trait-related general tendency to experience decision difficulties across a variety of situations” (Patalano et al., 2010, p. 353). It has been noted that indecisiveness is not specific to OCD (Thordarson et al., 2004), and in fact, indecisiveness has been shown to be associated with trait-anxiety (McNeil et al., 2016; Rassin and Muris, 2005), depression (Di Schiena et al., 2013; Rassin and Murris, 2005), and hoarding (Pushkarskaya et al., 2017). Therefore, as indecisiveness is not a symptom specific to OCD, it is not considered as a diagnostic criterion for OCD in the DSM-5. However, Nisticò et al. (2021) conducted a systematic review regarding decision making in OCD and used a meta-analytic approach to show that the impairment of decision making in OCD might constitute a trait feature of the disorder itself. Therefore, indecisiveness is not unique to OCD but is considered to be a common feature in obsessive-compulsive patients. In addition, a strong association between OC tendencies and indecisiveness has been shown in non-clinical groups (Frost and Shows, 1993; Rassin and Muris, 2005). In light of this background, and considering the purpose of the present study, which was to comprehensively measure OC tendencies experienced by university students including a non-clinical group, indecisiveness has been included as a factor in the scale.

For these reasons, a proper factor structure of OC tendencies would include a five-factor structure: checking, cleaning, obsessions, ordering, and indecisiveness. Wootton et al. (2015) pointed out that one of the most widely used scales of OC tendencies is the Obsessive-Compulsive Inventory-Revised (OCI-R; Foa et al., 2002), which has been used in many recent studies (Oren et al., 2018; Dar et al., 2019). Although the OCI-R includes the hoarding factor, it does not include indecisiveness. Angelakis et al. (2017) examined the factor structure of OCI-R in a non-clinical sample and showed that the conceptual fit of data was slightly better when hoarding was excluded.

In addition to the problems of factor structure, discrimination may be reduced in measuring OC tendencies because most precedent scales were based on clinical groups (Li and Shimoyama, 2008). In fact, when Yoshida et al. (1995) administered the Maudsley Obsessional-Compulsive Inventory—a scale used for OC tendencies (Pleva and Wade, 2006)—to a healthy group of Japanese individuals, the mean of the total score was low: 3.9 (90% Cl = 1.7–6.1, *range* = 0–30). Moreover, in the Padua Inventory, low mean scores for ordering and impulse were observed in addition to a distorted distribution: ordering (*M* = 2.99, *range* = 0–16, skewness = 1.36) and impulse (*M* = 6.97, *range* = 0–37, skewness = 1.46) (Sugiura and Tanno, 2000). Furthermore, in the Japanese version of the OCI-R (Koike et al., 2020), the floor effect was observed in the neutralizing and cleaning dimensions. Hence, established scales have been ineffective in measuring OC tendencies among university students in Japan due to problems with the factor structure and discrimination. The purpose of the present study is to develop a five-factor structure to measure OC tendencies commonly experienced by Japanese university students, and to examine the reliability and validity of the scale.

This study consists of two surveys. In Survey 1, we conducted an exploratory factor analysis (EFA) to examine the factor structure of OC tendencies and observed test-retest reliability, criterion-related validity, and convergent validity. We employed the Self-Rating Depression Scale (SDS) and the State-Trait Anxiety Inventory-Trait (STAI-T) as external criterion, following previous studies, to evaluate criterion-related validity (Li and Shimoyama, 2008). For convergent validity, the OCI-R was used. For test-retest reliability, the length of the interval between tests was approximately two weeks. In Survey 2, we conducted a confirmatory factor analysis (CFA) after administering the test on nonclinical sample of university students, different from those in Survey 1.

Survey 1 Exploratory factor analysis, test-retest reliability, and criterion-related and convergent validity of the University Students version of the OC tendencies scale.

## 2 Method

### 2.1 Participants

Data were collected in 2020 from Cross Marketing, a company in Tokyo, Japan that conducts online marketing research. A total of 216 participants were included in the analysis (99 males, 117 females). Their ages ranged from 18 to 26 years (*M* = 20.72, *SD* = 1.66).

### 2.2 Generation of items related to OC tendencies

We aimed to develop a scale using a five-factor model consisting of checking, cleaning, obsessions, ordering, and indecisiveness. We collected and modified items from established scales that dealt with these factors. Since these items have been piloted extensively, this piloting step was important for providing evidence of validity and reliability for the developed scale (Dörnyei and Taguchi, 2010). A literature review revealed that five existing scales have been commonly used for measuring OC tendencies and indecisiveness in Japan: the Japanese version of the Padua Inventory (J-PI; Sugiura and Tanno, 2000); the Chinese version of the OC tendencies scale (Li and Shimoyama, 2008); the OC tendencies scale (Ide et al., 1995); the Japanese version of the Maudsley Obsessional-Compulsive Inventory (Hosoba et al., 1992); and the Japanese version of the Frost Indecisiveness Scale (Sugiura et al., 2007). In selecting items to measure OC tendencies among university students, we excluded items that would be unfamiliar to young Japanese adults owing to cultural differences (e.g., weapons) or technological progress (e.g., public phones), as well as items that could be used in more than one way. This process of item selection was carried out through the collaboration of six graduate students specializing in clinical psychology. Afterwards, the selected items were adopted and revised by two authors via consensus.

### 2.3 Demographic variables

We assessed demographic variables, including age and gender (Table 1).

**Table 1:** Descriptive statistics of the university students in Surveys 1 and 2.

	Survey 1 (n = 216)			Survey 2 (n = 202)		
	All	Male	unspecified	All	Male	unspecified
Number	216	99	0	202	65	2
Age (years)	20.72 ± 1.66	20.81 ± 1.72	-	21.13 ± 1.82	21.32 ± 2.06	22.00 ± 0.00

### 2.4 OC tendencies

We assessed OC tendencies using the Obsessive-Compulsive Tendencies Scale (UOC tendencies scale), that was created using the procedure described above. The scale consists of 40 items with five subscales: obsessions, checking, cleaning, ordering, and indecisiveness. Participants rated each item on a 5-point Likert scale ranging from 1 (*not at all*) to 5 (*very much*).

### 2.5 Criterion-related validity

For criterion-related validity, we assessed depression using the Japanese version of the SDS (Fukuda and Kobayashi, 1973) and trait anxiety using the Japanese version of STAI-T (Shimizu and Imae, 1981). The SDS consists of 20 items. Participants rated each item on a 4-point Likert scale ranging from 1 (*never or rarely*) to 4 (*most or all of the time*). The STAI-T consists of 20 items. Participants rated each item on a 4-point Likert scale ranging from 1 (*almost never*) to 4 (*almost always*).

### 2.6 Convergent validity

To investigate convergent validity, we used the Japanese Version of the OCI-R (Koike et al., 2020), which consists of 18 items. Participants rated each item on a 5-point Likert scale ranging from 0 (*not at all*) to 4 (*extremely*). We expected a strong positive correlation between the OCI-R and the UOC tendencies scale.

### 2.7 Procedure

The questionnaire was administered using Google Forms and participants were recruited from the online panel database of Cross Marketing. Of the 216 total participants, 101 agreed to participate in the second test and complete the UOC tendencies scale two weeks after the first test.

### 2.8 Statistical analyses

All statistical analyses were conducted using R statistical software (Ver. 4. 0. 3; R Core Team, 2020) and SPSS version 25 (IBM Japan, Ltd.). First, we examined the Kaiser-Meyer-Olkin (KMO) index to evaluate data adequacy for factor analysis. Second, the *M* and *SD* of all items were calculated to reveal floor and ceiling effects. A floor effect was identified when the *M* minus *SD* value was less than one, and a ceiling effect was identified when the *M* plus *SD* value was more than five. Third, we determined the number of factors from eigenvalues, visual inspection of the scree plot, parallel analysis, Minimum Average Partial (MAP), and interpretability. After that, we conducted the exploratory factor analysis (maximum-likelihood estimation, promax rotation). Regarding factor loadings, we used .35 as a cutoff criterion for factor loading following Brown et al. (2012).

### 2.9 Ethical considerations

The participants were informed of the following four points before we administered the questionnaire: First, the data were statistically processed, and that the participants’ identifying information would be kept confidential. Second, the data would be used only in the current study. Third, participants could choose not to complete the questionnaire or could quit the study at any time. Fourth, they could withdraw their responses at any time. This study was approved by the ethics committee of the Kyushu University Faculty of Human-Environment Studies, Clinical Psychology Seminar. The study compiles with the ethical principles of the Declaration of Helsinki and the ethical rules established by the Japanese Psychological Association. In addition, informed consent was obtained for the study and their identities were anonymized.

## 3 Results

### 3.1 Factor structure of the UOC tendencies scale: EFA

First, we calculated the KMO index to examine the data adequacy for factor analysis. The overall value was 0.87, which was high, and factor analysis was judged to be applicable. Second, we examined floor and ceiling effects. Two items were found to have a floor effect, and these distributions were distorted. We considered these two items unfit for statistical analysis and excluded them.

The observed eigenvalues indicated an eight-factor structure with λ > 1 (9.33, 4.48, 2.44, 2.41, 1.63, 1.25, 1.20, and 1.09). However, after visual inspection of the scree plot, parallel analysis, and MAP, the criterion suggested the five-factor solution. Considering these results and interpretability, a five-factor solution was adopted in this study. Next, we conducted the exploratory factor analysis (maximum-likelihood estimation, promax rotation) for 38 items (Table 2). We excluded items with a factor loading that was lower than .35 or was high for multiple factors. As a result, we excluded 10 items and retained a five-factor model that consisted of 28 items. All Item-Remainder correlations were of high standards, and there were no items that improved the alpha coefficient significantly after deletion. Therefore, we did not exclude any other items. Following previous research, we named the first factor “checking,” the second factor “ordering,” the third factor “cleaning,” the fourth factor “indecisiveness,” and the fifth factor “obsessions.” The factor correlation was -.04∼.56 and Cronbach's alpha values for the five factors were sufficient (.79, .82, .78, .84, and .74, respectively).

**Table 2:** Factor loadings for exploratory factor analysis of the university students version of obsessive-compulsive tendencies Scale with promax rotation.

Item numbers	F1	F2	F3	F4	F5	*Mean*	*SD*	Correlation with remainder	Alpha after deletion
1. Checking									
21	**.83**	.13	-.01	-.19	-.04	3.01	1.31	.62	.74
39	**.76**	.12	-.08	-.20	.21	2.86	1.30	.67	.72
23	**.60**	-.20	.00	.22	-.09	3.53	1.12	.48	.77
13	**.42**	.02	.07	.18	-.08	3.36	1.04	.47	.77
29	**.42**	.30	-.08	.27	.11	3.05	1.34	.61	.74
7	**.39**	.02	.09	.00	.03	3.14	1.21	.39	.79
2. Ordering									
24	.14	**.83**	-.16	.01	.03	2.46	1.15	.71	.74
37	-.04	**.82**	.02	.01	.07	2.30	1.16	.72	.74
10	.05	**.63**	.23	.10	-.20	2.36	1.09	.59	.80
28	.03	**.61**	-.05	-.05	.15	2.61	1.25	.57	.82
3. Cleaning									
4	.04	.15	**.67**	.03	-.19	2.72	1.32	.56	.74
8	.15	-.07	**.66**	.08	-.09	3.32	1.19	.57	.74
3	.03	-.08	**.65**	.08	-.04	3.39	1.18	.54	.75
1	-.03	-.15	**.65**	-.15	.12	2.81	1.23	.53	.75
34	-.14	.08	**.51**	.00	.34	2.53	1.19	.53	.75
12	.00	.16	**.47**	-.07	.07	2.67	1.28	.46	.77
4. Indecisiveness									
31	.00	.03	-.02	**.79**	-.03	3.48	1.12	.67	.81
30	-.10	.20	-.04	**.77**	.14	3.10	1.20	.69	.80
2	.02	-.02	-.04	**.69**	-.09	3.33	1.28	.61	.82
32	-.16	-.01	.22	**.63**	.14	3.26	1.23	.60	.82
5	-.06	-.01	-.05	**.63**	.01	3.42	1.1	.55	.83
22	.05	-.06	-.02	**.61**	.07	3.22	1.21	.63	.82
5. Obsessions									
33	-.12	.08	.01	.10	**.69**	2.74	1.30	.55	.68
27	-.02	.09	-.03	-.06	**.57**	2.56	1.24	.43	.72
6	.09	.21	-.06	.22	**.52**	2.97	1.16	.55	.68
36	.02	.11	.06	-.01	**.49**	2.58	1.19	.42	.72
26	.12	-.02	-.08	.17	**.41**	3.19	1.13	.51	.70
9	.18	-.18	.09	.00	**.35**	3.41	1.18	.41	.72
Correlations between the factor						F2	F3	F4	F5

					F1	.25	.35	.56	.50
					F2		.31	-.04	.14
					F3			.26	.37
					F4				.55
									

### 3.2 Test-retest reliability

We calculated the Pearson's correlation to investigate the temporal stability of the UOC tendencies scale. The test-retest interval was two to three weeks. The reliability coefficient, which was the correlation between total scores in the UOC tendencies scale, was slightly lower (*r* = .68, *p* < .001). According to the scatter diagram, one participant was an outlier. Therefore, we performed the Smirnov-Grubbs test, which also revealed that one participant was an outlier (*p* < .01). We then excluded the data of that participant, and the resulting correlation coefficient showed an acceptable score (*r* = .73, *p* < .001). The reliability of the subscales of the UOC tendencies scale was .67 (checking), .61 (ordering), .79 (cleaning), .74 (indecisiveness), and .68 (obsessions).

### 3.3 Convergent and criterion-related validity

We calculated the sum of UOC tendencies scores and correlation coefficients with SDS, STAI-T, and OCI-R, to investigate convergent and criterion-related validity (Table 3). Both correlations were significantly positive, and the scores were .18 (*p* < .01), .39 (*p* < .001), and .52 (*p* < .001) for SDS, STAI-T, and OCI-R, respectively.

Survey 2 Confirmatory factor analysis of the UOC tendencies scale

**Table 3:** Convergent and criterion-related validity (Survey 1).

	Depression	STAI-T	OCI-R
UOC tendencies scale total	.18∗∗	.39∗∗∗	.52∗∗∗
Checking	.04	.22∗∗	.37∗∗∗
Ordering	-.10	-.11	.30∗∗∗
Cleaning	.14∗	.16∗	.37∗∗∗
Indecisiveness	.12	.45∗∗∗	.24∗∗∗
Obsessions	.36∗∗∗	.53∗∗∗	.51∗∗∗

∗∗∗*p* < .001. ∗∗*p* < .01. ∗*p* < .05.

## 4 Method

### 4.1 Participants

Data for CFA were collected from students by ASMARQ, a company in Tokyo, Japan that has been conducting online marketing research since 2020. A total of 202 participants were included in the analysis (65 males, 135 females, 2 unspecified). Their ages ranged from 18 to 29 years (*M* = 21.13, *SD* = 1.82).

### 4.2 Demographic variables

We assessed demographic variables, including age and gender (Table 1).

### 4.3 OC tendencies

We used the 40-item version of the UOC tendencies scale described in Survey 1 to assess OC tendencies.

### 4.4 Mental health

To measure the mental health of university students, we used the Japanese version of the Kessler 6-item Psychological Distress Scale (K6) (Furukawa et al., 2008). Participants rated each item on a 5-point Likert scale ranging from 1 (*never*) to 5 (*always*).

### 4.5 Statistical analyses

All statistical analyses were conducted using the same tools that were used in Survey 1. We conducted the CFA with a structural equation model. Although it is under debate which indices should be used, Brown (2006) did not include goodness-of-fit or the adjusted goodness-of-fit index due to their poor behavior in previous studies (e.g., Hu and Bentler, 1998; Marsh et al., 1988). Following Brown (2006), the following indicators were used: standardized root mean square residual (SRMR) with values ≤0.08, root mean square error of approximation (RMSEA) with values ≤0.06, and Tucker-Lewis index (TLI) and comparative fit index (CFI) with values ≥0.90. We used chi square divided by the degree of freedom with values <3 (Kline, 1998) as an additional indicator.

### 4.6 Procedure

The questionnaire was administered using Google Forms. All participants were recruited from an online panel database provided by ASMARQ.

## 5 Results

### 5.1 Factor structure: confirmatory factor analysis

We conducted the CFA (maximum likelihood method) to test the five-factor model for OC tendencies. The results showed that all path coefficients were significant at the 0.1% level (Table 4). The model fit was examined using the $\chi^{2}$/df, CFI, SRMR, TLI, and RMSEA. Our model fit results were as follows: $\chi^{2}$/df = 1.70; CFI = .911; TLI = .898; SRMR = .061; and RMSEA = .059. All model indicators were sufficient, except the TLI which was slightly below the criterion (≥.90). The five-factor model had sufficient internal reliability, with Cronbach's alphas varying between .81 and .87.

**Table 4:** Factor Loadings for Confirmatory Factor Analysis of the University Students version of Obsessive-Compulsive Tendencies Scale with Promax Rotation.

Item numbers	Standardizing Coefficient	*Mean*	*SD*
1. Checking			
29	.77	3.17	1.14
23	.72	3.62	1.04
13	.68	3.56	0.98
21	.63	3.34	1.16
39	.62	3.00	1.23
7	.55	3.09	1.11
2. Ordering			
28	.80	2.64	1.12
37	.79	2.72	1.15
10	.78	2.73	1.13
24	.69	2.78	1.10
3. Cleaning			
4	.68	3.05	1.29
8	.68	3.51	1.11
34	.66	2.89	1.13
1	.65	3.01	1.18
12	.62	2.90	1.26
3	.49	3.61	1.11
4.Indecisiveness			
30	.78	3.28	1.12
5	.77	3.53	1.10
31	.77	3.55	1.07
22	.73	3.24	1.13
32	.72	3.39	1.14
2	.53	3.36	1.22
5. Obsessions			
26	.73	3.39	1.04
36	.72	2.79	1.12
9	.66	3.68	1.03
33	.64	2.88	1.26
27	.64	2.72	1.29
6	.58	3.13	1.18

### 5.2 Descriptive statistics

We examined the descriptive statistics for each variable (Table 5). We also conducted the correlation analysis, which revealed that all subscales of the UOC tendencies scale were significantly correlated with K6 (Table 5).

**Table 5:** Means with 95% confidence intervals (Cls) and standard deviations for all variables and criterion-related validity and correlation with K6 (Survey 2).

Variables	*Mean*	*SD*	95%Cl	Correlation
UOC tendencies scale				
Total	95.06	19.41	92.39/97.74	.53∗∗∗
Checking	19.78	4.86	19.11/20.45	.38∗∗∗
Ordering	10.88	3.71	10.37/11.39	.23∗∗
Cleaning	18.97	5.11	18.27/19.68	.28∗∗∗
Indecisiveness	20.35	5.30	19.62/21.08	.39∗∗∗
Obsessions	18.59	5.15	17.88/19.30	.65∗∗∗
STAI				
Trait Anxiety	50.85	10.63	49.38/52.31	-
K6				
Mental Health	15.08	6.59	14.17/15.99	-

∗∗∗ *p*< .001. ∗∗*p* < .01. ∗*p* < .05.

## 6 General discussion

We developed a scale to assess OC tendencies by selecting items from multiple established scales to overcome the problem of changing the factor structure due to item reduction. The results showed that there was no reduction in the number of factors; therefore, we retained the five-factor model consisting of checking, ordering, indecisiveness, cleaning, and obsessions. Although twelve items were excluded, all adopted items were included in the expected factor. Regarding the number of factors related to OC tendencies, a four-factor structure (doubt and control/obsessions, checking, cleaning, and ordering) has been retained in many studies (Li and Shimoyama, 2008). Furthermore, some scales extract indecisiveness of healthy groups in Japan (Ide et al., 1995; Li and Shimoyama, 2008; Nishimura, 1997). Moreover, the five-factor structure has satisfactory construct validity as a scale of OC tendencies. Indecisiveness, which has not been measured adequately in established scales of OCD, such as the PI, OCI, Y-BOCS, and LOI, was extracted in this study. The *mean* of indecisiveness was the highest score among the five-factors. This might be due to cultural differences. Yates et al. (2010) found that Japanese university students were more indecisive compared to Chinese and European Americans and suggested that thoroughness might be an important cognitive mechanism among Japanese. In other words, it has been pointed out that in Japan, thorough analysis is encouraged during decision making. Therefore, cultural factors such as the encouragement of obsessive-compulsive behavior before making a decision may have contributed to the high level of indecisiveness. Although TLI was the only indicator that did not meet the criterion, the other model indices showed sufficient values, suggesting that the five-factor structure has an acceptable fit for the sample of Survey 2. The factor correlation in our study (-.04∼.56) was similar to that of previous research (Li and Shimoyama, 2008; .05∼.50).

All subscales of our UOC tendencies scale had a positive correlation to the K6. This result suggests that OC tendencies may be related to various psychological problems that need to be explored to improve the mental health of university students. As expected, the correlation between the scores on the UOC tendencies scale and the SDS, STAI-T, and OCI-R revealed small to medium coefficients (.18, .52, and .39, respectively). However, the correlation coefficient between indecisiveness and STAI-T (.45) is almost twice higher than between indecisiveness and OCI-R (.24). This association seems strong, even after accounting for the differences in the factor structure between the UOC tendencies scale and OCI-R. This implies that indecisiveness is not specific to OC tendencies, and is related to anxiety as well. Nevertheless, the UOC tendencies scale overall has sufficient criterion-related and convergent validity. These correlations between the scores of each factor of the UOC tendencies scale, and the SDS and STAI-T showed a pattern similar to previous research (Li and Shimoyama, 2008).

The test-retest reliability coefficient of the UOC tendencies scale was .73. Compared with the test-retest reliability of the previous scale measuring obsessive-compulsive symptoms, the result was somewhat lower (e.g., PI: .78). Among the five factors, the reliability of Ordering was the lowest. Ordering is closely related to incompleteness, and incompleteness has also been described as “*quite subtle and momentary emotional experiences”* (Davine et al., 2019, p. 482). This may have influenced the scores, which are likely to fluctuate over time. Furthermore, there is no clear standard regarding test-retest coefficients; however, according to Oshio (2016), the desirable mean test-retest correlation is .76 (95% CI = .70-.81). Therefore, the OC tendencies scale had acceptable reliability.

In conclusion, the UOC tendencies scale developed in this study had internal consistency, test-retest reliability, convergent validity, and criterion-related validity. As this scale is easy to administer among large groups, it is expected to measure OC tendencies of Japanese university students and contribute to mental health support for those who suffer from OC tendencies and related psychological problems (e.g., suicidal ideation, affinity for social withdrawal). Furthermore, the UOC tendencies scale can be used in empirical studies on OC tendencies among university students.

This study has certain limitations. First, the number of female participants was twice that of male participants. To investigate gender differences in further detail, the number of men and women should be matched. Second, the aim of this study was to develop a scale to assess the possible OC tendencies among university students. However, we could not provide suggestions to treat these OC tendencies. Third, indecisiveness had a stronger correlation with the STAI-T than the OCI-R. This result indicates that both OC tendencies and anxiety may strongly influence indecisiveness. Therefore, further research is needed to examine the interaction between both anxiety and OC tendencies regarding indecisiveness.

## Declarations

### Author contribution statement

Kenta Sashikata and Eiji Ozawa: Conceived and designed the experiments; Performed the experiments; Analyzed and interpreted the data; Contributed reagents, materials, analysis tools or data; Wrote the paper.

### Funding statement

This work was supported by 10.13039/501100004096Kyushu University (grant number: KYUDAI-KI-SHOGAKU-299).

### Data availability statement

The data that has been used is confidential.

### Declaration of interest's statement

The authors declare no conflict of interest.

### Additional information

No additional information is available for this paper.

## Appendix A Supplementary data

Supplementary file_V3 (1).docx Supplementary file_V3 (1).docx
