# Supplementary material for: Development of a scale to assess obsessive-compulsive tendencies among Japanese university students
Source: Heliyon. 2022 Jun 10;8(6):e09646. doi: 10.1016/j.heliyon.2022.e09646 (PMC9207615; doi:10.1016/j.heliyon.2022.e09646)
Supplement: Supplementary file_V3 (1).docx [file mmc1.docx]

$\lambda$Appendix

| University students version of Obsessive-Compulsive Tendencies scale for university students (UOC tendencies scale) | |
| --- | --- |
| Item number | Questions |
| Factor 1: Checking | |
| 21 | I sometimes have to return home to check things (e.g., door or windows, drawers, etc.) to make sure they are properly shut. |
| 39 | I sometimes check and recheck gas, water taps, and doors. |
| 29 | I sometimes keep on checking things more often than necessary. |
| 23 | I sometimes worry about losing my things or forgetting something. |
| 13 | I sometimes check several times when I do something. |
| 19 | I sometimes have to do things several times before I think they are properly done. |
| Factor 2: Ordering | |
| 37 | I feel uneasy when the things I see are not clean. |
| 24 | I have to keep things on the table clean. |
| 10 | I have to keep my things and room clean. |
| 28 | I feel uneasy when books are not lined up neatly on the bookshelf. |
| Factor 3: Cleaning | |
| 1 | I feel unclean when I accidentally bump into somebody in the train. |
| 34 | I find it difficult to touch something when I know it has been touched by someone. |
| 4 | I feel my hands are dirty after touching money. |
| 3 | I think even slight contact with my body (perspiration, saliva, urine, etc.) may contaminate clothes or somehow harm me. |
| 8 | If I touch something I think is dirty I immediately have to wash or clean myself. |
| 12 | I feel dirty when I touch animals and I immediately want to wash my hands or change my clothes. |
| Factor 4: Indecisiveness | |
| 2 | I find it difficult to make decisions when I see the menu and order or go shopping. |
| 22 | I am slow in making decisions. |
| 31 | I sometimes become anxious when I have to make decisions. |
| 30 | I sometimes find it difficult to make decisions even about trivial things. |
| 32 | I hate being in a situation where I have to decide something. |
| 5 | I sometimes worry about the decisions I have made. |
| Factor 5: Obsessions | |
| 33 | I sometimes worry about losing control and doing disturbing things. |
| 36 | I have feelings of distrust and doubt about most of my actions. |
| 26 | When I start thinking of certain things, I sometimes become obsessed with them. |
| 6 | I sometimes cannot pay attention to what is happening around me because my mind starts thinking certain things automatically. |
| 9 | When unpleasant thoughts come into my mind, I sometimes cannot get rid of them. |
| 27 | I sometimes worry, for no reason, that I have some disease. |

| University students version of Obsessive-Compulsive Tendencies scale for university students in Japanese (UOC tendencies scale) | |
| --- | --- |
| Item number | Questions |
| Factor 1: Checking | |
| 21 | ドアや窓，引き出しがきちんと閉まっているかどうか，確かめに戻る時がある。 |
| 39 | ガスや水道の栓，ドアの鍵などを何度かチェックすることがある。 |
| 29 | 必要以上に確認を繰り返すことがある。 |
| 23 | 何か物をなくしたのではないか，忘れ物をしたのではないかと思う時がある。 |
| 13 | 何かをしたときには，何度かチェックする。 |
| 19 | きちんとやれたと思うまでに，物事を何度か繰り返さなくてはならない時がある。 |
| Factor 2: Ordering | |
| 37 | 目に見えた物が片づいてないと気になる。 |
| 24 | テーブルの上に置いた物をきれいに片づけないと気がすまない。 |
| 10 | 自分の物や部屋をきれいに片づけないと気がすまない。 |
| 28 | 本棚にある本がきれいに並んでいないと気がすまない。 |
| Factor 3: Cleaning | |
| 1 | 電車などで接触したとき，汚いと感じる。 |
| 34 | 誰かが前にさわっていた物に触れるのは嫌だと感じる。 |
| 4 | お金に触ると，手が汚れたと感じる。 |
| 3 | 汗や唾や小便などに少しでも触れると，服が汚れたとか，私にとって何らかの害になるだろうと思う。 |
| 8 | 汚いと思う物に触ったら，すぐにきれいにしないと気がすまない。 |
| 12 | 動物に触ったら汚い感じがして，すぐに手を洗ったり着替えたりしたくなる。 |
| Factor 4: Indecisiveness | |
| 2 | メニューをみて注文する時，または買い物する時，なかなか決められない。 |
| 22 | 物事を決めるのが遅い。 |
| 31 | 決断するとき，不安になることがある。 |
| 30 | 小さなことでも決心するのは難しいと感じる時がある。 |
| 32 | 何かを決定するような状況に置かれるのは嫌いだ。 |
| 5 | 一度決めたことを，後になって悩んだりする。 |
| Factor 5: Obsessions | |
| 33 | 自分をコントロールできなくなって，困ったことをしてしまうのではないかと心配になる時がある。 |
| 36 | 自分がすることの多くに不審や疑問を抱いてしまう。 |
| 26 | あることについて考え始めるとそれに取りつかれてしまう時がある。 |
| 6 | 頭が勝手にものを考えて，自分の周りで起こっていることに注意が向けられない時がある。 |
| 9 | 不愉快な考えが頭に浮かぶと，そのことが頭から離れない時がある。 |
| 27 | 自分の体の具合が悪いのではないかと，理由もなく気に病む時がある。 |
